# Non-contrast dual-energy CT virtual ischemia maps accurately estimate ischemic core size in large-vessel occlusive stroke

**DOI:** 10.1038/s41598-021-85143-3

**Published:** 2021-03-24

**Authors:** Dylan N. Wolman, Fasco van Ommen, Elizabeth Tong, Frans Kauw, Jan Willem Dankbaar, Edwin Bennink, Hugo W. A. M. de Jong, Lior Molvin, Max Wintermark, Jeremy J. Heit

**Affiliations:** 1 Department of Neuroimaging and Neurointervention, Stanford University Hospital, 300 Pasteur Drive, Room S-047, Stanford, CA 94305 USA; 2 Department of Radiology, University Medical Center Utrecht, Utrecht, The Netherlands; 3 Department of Radiology, Stanford University Hospital, 300 Pasteur Drive, Room S-047, Stanford, CA 94505 USA

**Keywords:** Diagnosis, Neurological disorders, Neurovascular disorders, Stroke, Medical imaging, Brain imaging

## Abstract

Dual-energy CT (DECT) material decomposition techniques may better detect edema within cerebral infarcts than conventional non-contrast CT (NCCT). This study compared if Virtual Ischemia Maps (VIM) derived from non-contrast DECT of patients with acute ischemic stroke due to large-vessel occlusion (AIS-LVO) are superior to NCCT for ischemic core estimation, compared against reference-standard DWI-MRI. Only patients whose baseline ischemic core was most likely to remain stable on follow-up MRI were included, defined as those with excellent post-thrombectomy revascularization or no perfusion mismatch. Twenty-four consecutive AIS-LVO patients with baseline non-contrast DECT, CT perfusion (CTP), and DWI-MRI were analyzed. The primary outcome measure was agreement between volumetric manually segmented VIM, NCCT, and automatically segmented CTP estimates of the ischemic core relative to manually segmented DWI volumes. Volume agreement was assessed using Bland–Altman plots and comparison of CT to DWI volume ratios. DWI volumes were better approximated by VIM than NCCT (VIM/DWI ratio 0.68 ± 0.35 vs. NCCT/DWI ratio 0.34 ± 0.35; P < 0.001) or CTP (CTP/DWI ratio 0.45 ± 0.67; P < 0.001), and VIM best correlated with DWI (r_VIM_ = 0.90; r_NCCT_ = 0.75; r_CTP_ = 0.77; P < 0.001). Bland–Altman analyses indicated significantly greater agreement between DWI and VIM than NCCT core volumes (mean bias 0.60 [95%AI 0.39–0.82] vs. 0.20 [95%AI 0.11–0.30]). We conclude that DECT VIM estimates the ischemic core in AIS-LVO patients more accurately than NCCT.

## Introduction

Dual-energy computed tomography (DECT) scanners acquire CT images at two different tube voltages, which allows for improved contrast resolution, image noise and beam hardening artifact reduction, and reconstruction of source data into images which separate individual tissue components, such as water, iodine, and fat on the basis of differential beam attenuation^1,2^. DECT techniques have been used following AIS-LVO patient treatment by endovascular thrombectomy (ET) to differentiate normal post-procedural iodine contrast presence from post-ET intracranial hemorrhage and to improve the visualization of cerebral ischemia when obscured by residual parenchymal contrast opacification^3,4^. Previously reported custom DECT postprocessing algorithms have suggested improved detection of cerebral ischemia by highlighting areas of cytotoxic edema after material separation using basis sets of fat, gray matter, and calcium, or air, iodine, and water, however these techniques have been technically limited and complex to interpret, or were not compared against reference standard DWI images^5,6^. As accurate ischemic core delineation is critical in the triage of AIS-LVO patients to ET, CT techniques capable of superior core estimation are of high clinical relevance^7–9^.

We hypothesized that postprocessing of non-contrast DECT using an automated material decomposition algorithm to generate Virtual Ischemia Maps (VIM) may more accurately identify and estimate the ischemic core in AIS-LVO patients compared to conventional NCCT, and may be similar to ischemic core estimates derived from CTP techniques. Relative to DWI-derived final core infarct volumes, we primarily evaluated the accuracy of DECT-derived VIM reconstructions in delineating ischemic core volumes on baseline imaging of AIS-LVO patients undergoing ET triage. Secondarily, we evaluated the qualitative accuracy of ischemic core estimation of VIM and NCCT reconstructions through multi-reader provision of Alberta Stroke Program Early CT Scores (ASPECTS) relative to a consensus DWI-ASPECTS.

## Methods

### Patients

This study complied with the Health Insurance Portability and Accountability Act and was approved by the Stanford University Institutional Review Board, which waived the need for written informed consent (Protocol 51077; IRB 61). We performed a retrospective cohort review of consecutive AIS-LVO patients who underwent DECT imaging triage for ET from 9/2018–8/2019. Patient demographic and treatment data were identified from a prospectively maintained AIS-LVO database at our comprehensive stroke center. A majority of AIS-LVO patients at our institution arrive by transfer from spoke hospitals and undergo immediate pre-procedural MRI, while inpatient or emergency department AIS patients undergo CT at the nearest available scanner. As such, not all patients undergo imaging at a DECT capable scanner, and technologists are instructed to perform single-energy CT if patient body habitus or motion are suspected to limit image quality on our institutional dual-source DECT.

Inclusion criteria were: baseline DECT, age > 18 years, presentation within 24 h of symptom onset, anterior circulation large vessel occlusion (ICA, M1- or proximal M2- segment of the middle cerebral artery), and follow-up MRI with DWI within 24 h of DECT. To minimize errors in ischemic core estimation between baseline DECT and follow-up reference standard DWI, only patients with matched perfusion-ischemic core profiles on baseline imaging or those with successful revascularization (Thrombolysis in Cerebral Ischemia [TICI] grade 2b, 2c, or 3) after ET were included^8,10–12^. Matched perfusion-ischemic core profiles were defined as patients with a < 15 mL difference between CBF < 30% and Tmax > 6 s volumes and mismatch ratio < 1.8.

Exclusion criteria included technical failure of perfusion imaging, excessive motion artifact, and absence of core infarction on follow-up MRI. The patient selection workflow and reasons for exclusion are summarized in Supplemental Figure 1.

### Imaging technical details

All patients underwent DECT on a dual-source SOMATOM Definition Flash (Siemens Healthcare, Munich, Germany). The CT protocol included a non-contrast head CT, CT perfusion (CTP), and CT angiography of the head and neck. All DECT protocols were developed to maintain strict dose neutrality with prior conventional CT protocols at our institution. Scan parameters were: tube voltages of 80-kVp and tin-filtered 140-kVp, tube currents of 640 and 320 mAs, pitch 1.0, beam collimation 40 × 0.6 mm, rotation time 1.0 s, matrix size 512 × 512, and slice thickness 3.0 mm. Tin filter was applied to the high-voltage tube to improve spectral separation. Images were reconstructed using a medium smoothing Q34s kernel, and conventional appearing blended NCCT images were generated to match an effective 120-kVp image, as is the clinical standard at our institution.

DECT VIM images were generated from non-contrast source data in Syngo.via (Siemens Healthcare, Munich, Germany) using the three-material Brain Hemorrhage decomposition module normally used to generate virtual non-contrast images, using basis materials of hemorrhage, CSF, and iodine. The following manufacturer default material definitions were used: Low Energy: Hemorrhage 80 HU, CSF 5 HU; High Energy: hemorrhage 75 HU, CSF 0 HU; and relative CM 2.0. Algorithm parameters included: minimum and maximum thresholds − 20 HU and 3071 HU, CM cutoff − 50 HU, and resolution 2. Resolution enhancement, organ contour enhancement, and iodine beam hardening correction were enabled.

Whole-brain CTP was performed as previously described and CTP-derived ischemic core volumes were automatically calculated using RAPID (iSchemaView, Menlo Park, CA), which identifies the ischemic core as a 70% reduction in cerebral blood flow (CBF) relative to the contralateral normal hemisphere, termed CBF < 30%^13^. CBF < 38% volumes were additionally collected as these maps have shown superior accuracy to final DWI infarct volumes, but are not routinely used in AIS-LVO patient triage to avoid inappropriate exclusion from treatment^14^. Whole-brain DWI-MRI was performed for reference standard final core infarct assessment, using either a 1.5 T GE Signa or 3.0 T GE MR750 MRI scanner with an eight-channel GE HR brain coil (GE Healthcare, Milwaukee, Wisconsin). Axial DWI imaging parameters included: TR 6000 ms, TE 78.2 ms, b-values: 0 and 1000, flip angle 90°, and 5 mm slice thickness.

### Imaging analysis

Anonymized neuroimaging data were placed into an Osirix database (Pixmeo, Bernex, Switzerland) for analysis. Imaging readers had full control in modifying window width and level for all images and were blinded to clinical, thrombectomy, and MRI data.

Ischemic core volumes were manually segmented as ROIs on VIM, NCCT, and DWI images by DNW, a neuroradiology fellow, and verified by JJH, a neuroradiologist with 7-years of experience. CT regions of pathological parenchymal hypoattenuation were segmented, which required loss of gray-white differentiation, an absence of local volume loss, and conformation to an anterior circulation vascular territory. DWI regions of infarction were segmented on the basis of hyperintensity on high b-value images, and segmentation was performed only within the penumbral volume. Volumes were calculated from manual ROI segmentation using a custom Osirix plug-in^15^.

Alberta Stroke Program Early CT Score (ASPECTS) was assigned for all studies by three readers (JJH; DNW; ET, a neuroradiologist with 4-years of experience), each provided with baseline VIM images followed by baseline conventional NCCT images after a 6-week interval to minimize recall bias. Consensus DWI-ASPECTS, determined after an additional 6-week interval by the same readers, was used as the reference standard.

### Statistical analysis

The primary outcome measure was agreement between VIM, NCCT, and CTP ischemic core volumes compared to the reference standard DWI volumes. Secondary outcome measures were the accuracy of VIM- and NCCT-ASPECTS relative to consensus DWI-ASPECTS, and inter-reader agreement for the ASPECTS analysis. Both per-reader and pooled analyses were performed.

Pairwise comparisons of all ischemic core volumes derived from VIM, NCCT, CTP, and DWI were performed using the Mann–Whitney U test, and ASPECTS comparisons were performed using the Wilcoxon signed rank test. Normalized ischemic core volumes were calculated as the fraction of the CT volume divided by the DWI volume. Spearman-rank correlation coefficients were calculated for comparisons of ASPECTS, while Pearson correlation coefficients were calculated for ischemic core volume comparisons following logarithmic volume correction for non-Gaussian data distribution and a log(x + 1) constant correction to prevent exclusion of zero values. Modified Bland–Altman analyses, subsequently referred to as Bland–Altman analyses for simplicity, were used to assess for volume and ASPECTS agreement between NCCT, VIM, and reference DWI, both before and after logarithmic correction of non-Gaussian data. Bland–Altman analyses were modified such that DWI-derived data were considered the reference standard, and CT-derived data were treated as the dependent test^16,17^. Bland–Altman plots are presented following logarithmic data regression to correct for non-Gaussian data distribution, as described in the Supplemental Methods. Mean bias values and 95% agreement intervals (95%AI) were reported, with significant differences between data sets determined by absence of overlap between the agreement intervals. No P-values are provided for Bland–Altman analyses as they may inadequately reflect clinically meaningful differences between measurement methods or affirm spurious correlations^16^. Bland–Altman analyses were selected as a better representation of a reader’s subjective evaluation versus other volumetric comparisons, such as a Dice coefficient, which may additionally introduce quantitation error from transformation and co-registration of CT to DWI images. Inter-reader reliability for VIM- and NCCT-ASPECTS were assessed using Fleiss’ κ test and the intraclass correlation coefficient (ICC).

Subgroup analyses of ischemic core volumes and ASPECTS agreement between VIM, NCCT, and DWI were performed as a function of time from last known normal (LKN) to CT imaging. Patients imaged within the early-window (≤ 6 h) were compared against those in the late-window (> 6 h) to determine if VIM performance varied between windows.

Data were analyzed in Matlab R2020b (Mathworks, Natick, Massachusetts) and SPSS 25.0 (IBM, Armonk, New York) by FVO and DNW. Statistical significance was set at α = 0.05.

## Results

### Baseline patient characteristics

Twenty-four patients (46% female, 64.3 ± 15.0 years) who underwent DECT triage for AIS-LVO were included (Supplemental Figure 1). Patient demographic data and baseline imaging characteristics are summarized in Table 1. LVO were localized to: ICA (5/24; 21%), M1 segment of the MCA (11/24; 46%), tandem ICA-M1 segment (2/24; 8%), and proximal M2 segment of the MCA (6/24; 25%). The median time from LKN to CT was 7.5 (IQR 1.5–14.5) hours, and the median time from CT to MRI was 9.1 (IQR 2.3–14.5) hours. Eleven patients (46%) presented in the early time window and underwent DECT ≤ 6 h from last known normal time. The median presentation NIHSS was 16 (IQR 7–20). Eight patients (33%) underwent emergent ET with all achieving TICI 2b (1/8; 13%), TICI 2c (3/8; 38%), or complete (TICI 3 [4/8; 50%]) revascularization.

**Table 1:** Patient demographics and baseline imaging characteristics.

	All patients	Early-window (≤ 6 h)	Late-window (> 6 h)
	(n = 24)	(n = 11)	(n = 13)
Sex (% female)	11/24 (46%)	7/11 (64%)	4/13 (31%)
Mean age (years ± st.dev.)	64.3 ± 15.0	65.8 ± 15.6	63.0 ± 15.0
Median presenting NIHSS [IQR]	16 [7–20]	19 [11–20]	11 [7–19]
**LVO location (%)**			
ICA	5/24 (21%)	4/11 (36%)	1/13 (8%)
M1	11/24 (46%)	4/11 (36%)	7/13 (54%)
Tandem ICA-M1	2/24 (8%)	1/11 (9%)	1/13 (8%)
Proximal M2	6/24 (25%)	2/11 (18%)	4/13 (31%)
Median LKN to CT (hours [IQR])	7.5 [1.5–14.5]	1.5 [0.9–2.4]	14.1 [9.7–17.2]
Median CT to MR (hours [IQR])	9.1 [2.3–14.5]	7.0 [1.4–11.4]	12.2 [5.4–15.4]
**Thrombectomy (%)**	8/24 (33%)	3/11 (27%)	5/13 (38%)
TICI 2b	1/8 (13%)	0/3 (0%)	1/5 (20%)
TICI 2c	3/8 (38%)	1/3 (33%)	2/5 (40%)
TICI 3	4/8 (50%)	2/3 (66%)	2/5 (40%)
Mean LKN to revascularization (hrs ± st.dev.)	9.6 ± 6.6	3.0 ± 2.1	13.6 ± 4.5
Mean CT to revascularization (hrs ± st.dev.)	1.5 ± 1.0	1.6 ± 1.5	1.5 ± 0.9

### Quantitative ischemic core volume assessment

**Figure 1:**
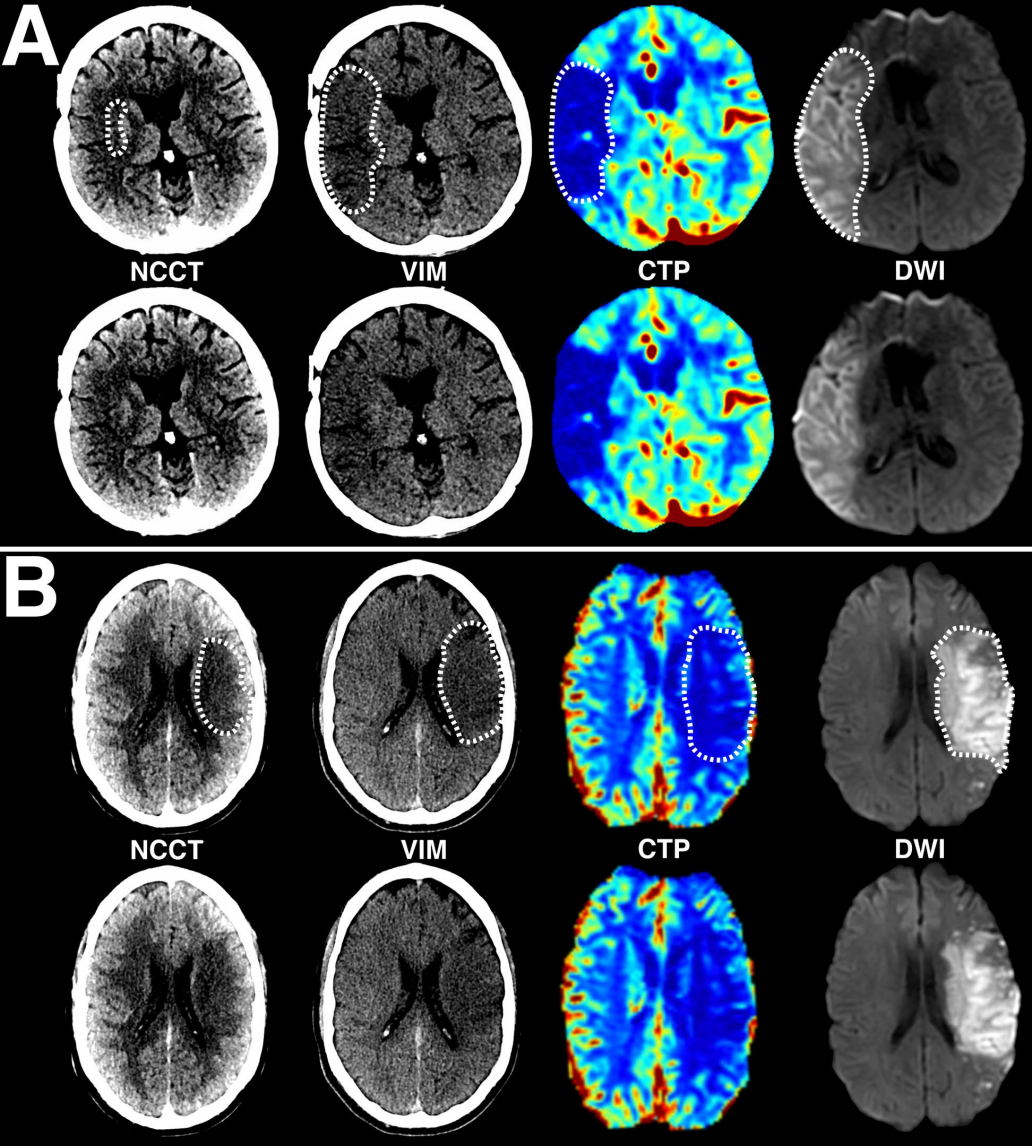
Examples of ischemic core conspicuity compared between CT and DWI methods. (**A**) Example case of a 49-year-old female presenting in the early-window with an occlusion of the proximal M1 segment of the right middle cerebral artery and a large core infarct. Please note that this patient had a distant history of right-sided craniotomy for anterior communicating artery aneurysm clipping, with evidence of the prior craniotomy seen on the CT images, and susceptibility artifact from the aneurysm clip visible on the DWI image adjacent to the frontal horn of the right lateral ventricle. (**B**) Additional example case of a 43-year-old male presenting in the late-window with an occlusion of the proximal M1 segment of the left middle cerebral artery and a large core infarct. For both patients, axial non-contrast CT (NCCT), virtual ischemia map (VIM), CT perfusion cerebral blood flow map (CBF) and diffusion-weighted MRI (DWI) images are presented. Note the increased conspicuity of the ischemic core on the axial non-contrast VIM series relative to the NCCT, using the same window width (40) and level (25) settings for each image. Within the same territory, reduced CBF and corresponding diffusion restriction on subsequent MRI confirm the territory of infarction. All regions of visible ischemic core are outlined by hashed white lines in the top row for each patient, with unannotated versions provided in the bottom row. Postprocessed DECT and perfusion images were automatically generated using Syngo.via (Siemens Healthcare, Munich, Germany) and RAPID (iSchemaView, Menlo Park, CA), respectively.

Ischemic core conspicuity prior to ET was compared between VIM, NCCT, and CTP (Fig. 1 and Table 2 [upper panel]). Normalized VIM ischemic core volumes more closely approximated DWI core volumes when compared to conventional NCCT (VIM/DWI ratio 0.68 ± 0.35 vs. NCCT/DWI ratio 0.34 ± 0.35; P < 0.001) or CTP core estimates (CBF < 30%/DWI ratio 0.45 ± 0.67, P < 0.001; and CBF < 38%/DWI ratio 0.52 ± 0.62; P = 0.06). Normalized NCCT ischemic core volumes and CTP core volumes did not significantly differ from each other, and less accurately approximated DWI core volumes (NCCT/DWI ratio 0.34 ± 0.35 vs. CBF < 30%/DWI ratio 0.45 ± 0.67, P = 0.12; and CBF < 38%/DWI ratio 0.52 ± 0.62, P = 0.66). Relative to DWI, VIM tended to underestimate absolute ischemic core volumes (74.8 ± 103.5 mL vs. 39.7 ± 39.3 mL; P = 0.26), but to a lesser degree than those volumes estimated from NCCT (18.6 ± 22.4 mL; P = 0.004) or CBF < 30% (34.0 ± 68.8 mL; P = 0.003), and similar to CBF < 38% volumes (41.6 ± 72.3 mL; P = 0.02). However, automatically derived CBF < 30% volumes were skewed by software segmentation errors (9/24 patients erroneously segmented as 0 mL, all with final DWI core volumes of ≤ 30 mL), and over-estimation of several cases using CBF < 38% volumes (3/24 patients with estimated core ratios > 1.25). Pairwise comparisons of absolute NCCT and CTP volumes, and VIM and CBF < 38% volumes did not significantly differ, while VIM and CBF < 30% and VIM and NCCT volumes did significantly differ (Table 2).

**Table 2:** Overall and time-dichotomized ischemic core volume and ASPECTS comparisons.

Overall core infarct volume and ASPECTS comparisons							
Overall ischemic core volume comparisons			Statistical mean ischemic core volume comparisons		Statistical normalized ischemic core comparisons		
	Mean Ischemic Core Volume (mL)	Mean normalized ischemic core volume against DWI		P-value		P-value	
			VIM vs. NCCT	< 0.001	VIM vs. NCCT	< 0.001	
Manual NCCT	18.6 ± 22.4	0.34 ± 0.35	VIM vs. CBF < 30%	0.02			
Manual VIM	39.7 ± 39.3	0.68 ± 0.35	VIM vs. CBF < 38%	0.22	VIM vs. CBF < 30%	< 0.001	
Automatic CBF < 30%	34.0 ± 68.8	0.45 ± 0.67	VIM vs. DWI	0.26			
Automatic CBF < 38%	41.6 ± 72.3	0.52 ± 0.62	NCCT vs. CBF < 30%	0.19	VIM vs. CBF < 38%	0.06	
			NCCT vs. CBF < 38%	0.93			
Manual DWI	74.8 ± 103.5	NA	NCCT vs. DWI	0.004	NCCT vs. CBF < 30%	0.12	
			CBF < 30% vs. DWI	0.003	NCCT vs. CBF < 38%	0.66	
			CBF < 38% vs. DWI	0.02			

Pooled ASPECTS comparisons							
	Mean ASPECTS ± SD	P-value	Fleiss’ k	ICC			
Consensus DWI ASPECTS	4.8 ± 2.4						
Pooled NCCT ASPECTS	7.6 ± 2.0	< 0.001	0.17	0.62 (0.40–0.80)			
Pooled VIM ASPECTS	6.4 ± 2.5		0.23	0.64 (0.43–0.81)			

Time-dichotomized core infarct volume and ASPECTS comparisons							
Time-dichotomized ischemic core volume comparisons							
	Early-window (n = 11)		Late-window (n = 13)		P-value		
Manual NCCT	13.0 ± 17.4 mL		23.3 ± 25.7 mL		0.2		
Manual VIM	41.2 ± 47.6 mL		38.5 ± 32.7 mL		0.64		
Manual DWI	86.5 ± 129.2 mL		64.9 ± 80.1 mL		0.52		
Normalized NCCT	0.20 ± 0.14	[< 0.001]	0.45 ± 0.35	[< 0.001]	0.11		
Normalized VIM	0.60 ± 0.32		0.75 ± 0.37		0.33		
Normalized VIM-NCCT	0.40 ± 0.25		0.30 ± 0.30		0.22		

Time-dichotomized ASPECTS comparisons							
	Early-window (n = 11)			Late-window (n = 13)			P-value
	Mean ASPECTS		ICC [IQR]	Mean ASPECTS		ICC [IQR]	
Consensus DWI ASPECTS	4.8 ± 2.7			4.8 ± 2.4			
Pooled NCCT ASPECTS	8.3 ± 1.7	[ < 0.001]	0.65 (0.32–0.88)	7.1 ± 2.2	[ < 0.001]	0.64 (0.34–0.86)	0.01
Pooled VIM ASPECTS	6.9 ± 2.5		0.57 (0.21–0.84)	6.0 ± 2.5		0.61 (0.30–0.84)	0.18

**Figure 2:**
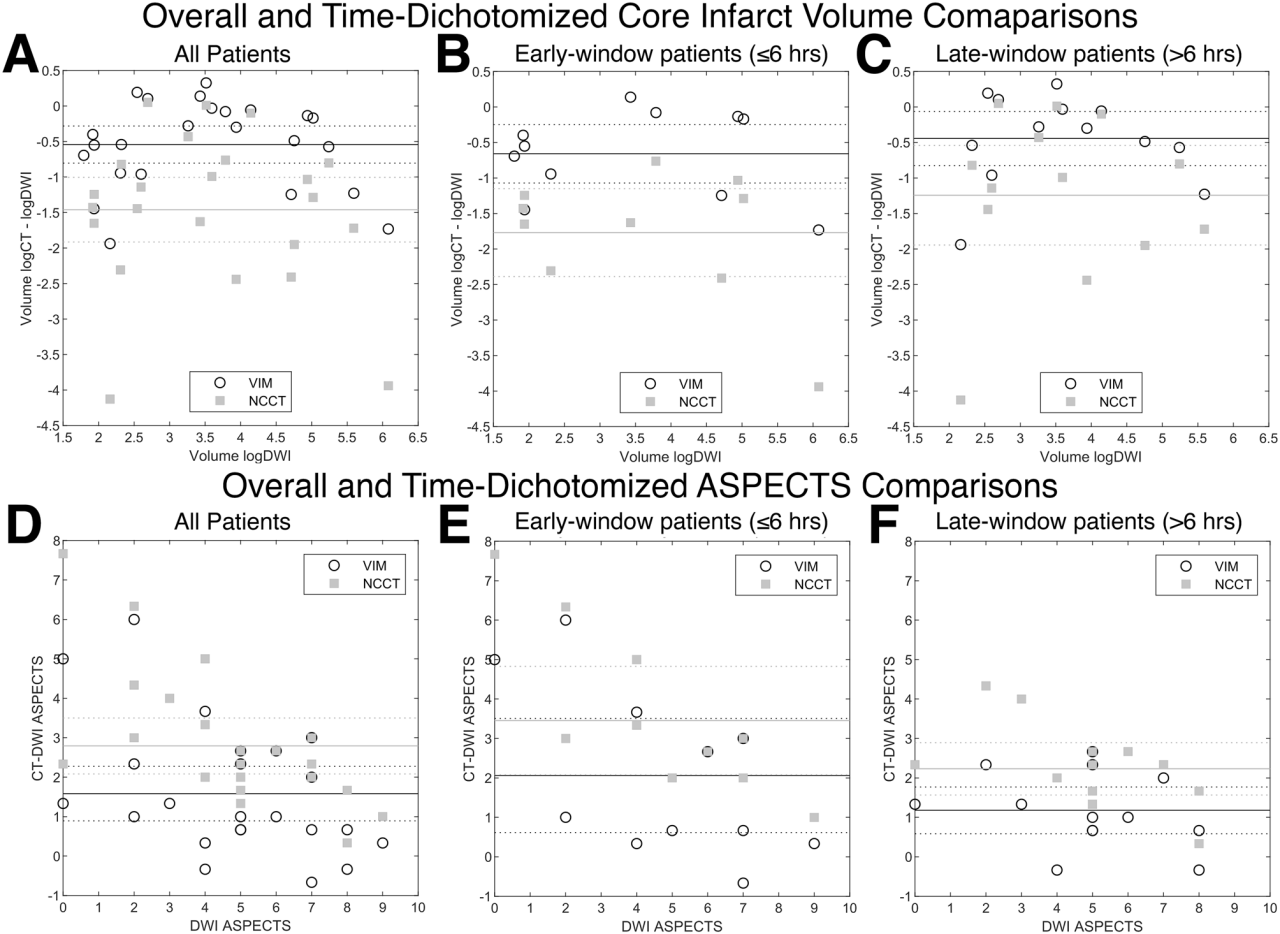
Overall and time-dichotomized Bland–Altman analyses of ischemic core volume and ASPECTS agreement. (**A**–**C**) Modified Bland–Altman analyses of ischemic core volumes are presented relative to reference standard DWI infarct volumes. In order to correct for a non-Gaussian distribution of volume data, logarithmically corrected VIM and NCCT volumes minus the logarithmically corrected DWI volumes are plotted against the logarithmically corrected DWI volumes for all patients (**A**), and for patients in the early- (**B**) and late-windows (**C**). Please refer to Supplemental Figure 2 for non-logarithmically corrected plots. (**D**–**F**) Modified Bland–Altman analyses of CT-ASPECTS are presented as the difference between VIM- or NCCT-ASPECTS and the corresponding consensus DWI-ASPECTS for all patients (**D**), and for patients in the early- (**E**) and late-windows (**F**). Solid lines indicate the mean bias value for each measurement, and hashed lines indicate the 95% agreement interval limits. Values closer to 0.0 indicate greater agreement with the reference DWI volume for (**A**–**C**), or greater agreement with the consensus DWI-ASPECTS for (**D**–**F**). Data were processed in SPSS 25.0 (IBM, Armonk, NY) and Matlab R2020b (Mathworks, Natick, MA), with the latter software used for graph generation.

CT-derived ischemic core volumes were compared against corresponding reference DWI volumes, and VIM best correlated with DWI (r_VIM_ = 0.90; r_NCCT_ = 0.75; r_CBF<30%_ = 0.77; r_CBF<38%_ = 0.61; P < 0.001) using the Pearson correlation coefficient of logarithmically corrected core volumes. Volume agreement was then assessed by Bland–Altman analyses comparing normalized CT volumes against reference DWI volumes in both logarithmically corrected and uncorrected plots (Fig. 2A, Supplemental Figure 2A). Mean bias values for normalized and logarithmically corrected VIM volumes were 0.68 (95%AI 0.53 to 0.83) and − 0.54 (95%AI − 0.81 to − 0.28), while bias values for NCCT were 0.34 (95%AI 0.21 to 0.46) and − 1.46 (95%AI − 1.92 to − 1.01), respectively. The VIM ischemic core volume mean bias values were closer to agreement with the DWI volumes than the NCCT, and corresponding agreement intervals were non-overlapping, indicating a significant difference.

### ASPECTS analysis

Pooled and individual reader ASPECTS results are shown in Table 2 (upper panel) and Supplemental Table 1. There was a significant difference in ischemia assessment between VIM- and NCCT-ASPECTS (6.4 ± 2.5 vs. 7.6 ± 2.0; P < 0.001). Pooled VIM-ASPECTS (6.4 ± 2.5) more closely agreed with consensus DWI-ASPECTS (4.8 ± 2.4; 71% accuracy) compared to NCCT-ASPECTS (7.6 ± 2.0; 65% accuracy). Reader experience was not correlated with accuracy. VIM- and NCCT-ASPECTS were plotted against corresponding consensus DWI-ASPECTS, with a similar Spearman correlation coefficient between VIM and DWI (ρ_VIM_ = 0.74; ρ_NCCT_ = 0.71; Supplemental Figure 3). A Bland–Altman analysis plotting the difference between CT-ASPECTS and DWI-ASPECTS was performed, with values closer to 0.0 indicating greater agreement (Fig. 2D). Mean bias values for the difference between VIM-ASPECTS (1.58; 95%AI 0.89–2.28) or NCCT-ASPECTS (2.79; 95%AI 2.08–3.50) and DWI-ASPECTS trended toward superior accuracy with VIM; however, the agreement intervals remained overlapping, indicating a non-significant difference. ASPECTS interrater agreement was similar for VIM (κ = 0.23; ICC = 0.64 [0.43–0.81]) and NCCT (κ = 0.17; ICC = 0.62 [0.40–0.80]).

### Ischemic core assessment in the early- and late-windows

The conspicuity of the ischemic core on CT may be influenced by the time since patients were LKN. To determine if the accuracy of VIM differed depending upon LKN time, patients were dichotomized into early (≤ 6 h) and late (> 6 h) time windows (Table 1). Forty-six percent (11/24) of patients presented early with a median LKN time of 1.5 h (IQR 0.9–2.4) and a median time from CT to MRI of 7.0 h (IQR 1.4–11.4). Fifty-four percent (13/24) of patients presented late with a median LKN time of 14.1 h (IQR 9.7–17.2) and a median time from CT to MRI of 12.2 h (IQR 5.4–15.4).

Ischemic core volume and ASPECTS results dichotomized by time window are shown in Table 2 (lower panel) and Supplemental Table 2, and normalized ischemic core volume and ASPECTS agreement are presented as a function of time in Supplemental Figure 4. Normalized VIM and NCCT core volumes significantly differed in both early (0.60 ± 0.32 vs. 0.20 ± 0.14; P < 0.001) and late (0.75 ± 0.37 vs. 0.45 ± 0.35; P < 0.001) patients; VIM core assessment was more accurate in both time windows. However, in early-window patients, the difference between VIM and NCCT core estimates (0.40 ± 0.25) were 25% larger than in late-window patients (0.30 ± 0.30; P = 0.22). In the early-window, Bland–Altman analyses demonstrated mean bias values for normalized and logarithmically corrected VIM volumes of 0.60 (95%AI 0.39 to 0.82) and − 0.66 (95%AI − 1.07 to − 0.25), while bias values for NCCT were 0.20 (95%AI 0.11 to 0.30) and − 1.77 (95%AI − 2.39 to − 1.15), respectively (Supplemental Figure 2B, Fig. 2B). Early-window VIM core volume bias values indicated greater agreement with the DWI volumes than NCCT, and corresponding agreement intervals were non-overlapping. In the late-window, mean bias values for normalized and logarithmically corrected VIM volumes were 0.75 (95%AI 0.52 to 0.97) and − 0.44 (95%AI − 0.83 to − 0.06), while bias values for NCCT were 0.45 (95%AI 0.23 to 0.67) and − 1.24 (95%AI − 1.95 to − 0.54), respectively (Supplemental Figure 2C, Fig. 2C). Late-window VIM core volume bias values showed a trend toward superior DWI volume estimation over NCCT, though overlapping agreement intervals indicated a non-significant difference between methods in this subgroup.

We then compared ischemic core determination by ASPECTS in the early- and late-windows. Relative to DWI-ASPECTS (early-window 4.8 ± 2.7 and late-window 4.8 ± 2.4), pooled VIM-ASPECTS was more accurate than NCCT-ASPECTS in both the early- (6.9 ± 2.5 vs. 8.3 ± 1.7; P < 0.001) and late-windows (6.0 ± 2.5 vs. 7.1 ± 2.2; P < 0.001; Table 2 [lower panel]). VIM-ASPECTS was more accurate than NCCT-ASPECTS for all readers in the early-window (P = 0.004, 0.03, 0.02), but only remained significant for two of three readers in the late-window (P = 0.02, 0.004, and 0.07; Supplemental Table 1). Individual reader accuracy was similar relative to DWI-ASPECTS, with improved accuracy for all readers using VIM images (Supplemental Table 1). Bland–Altman analyses plotting the difference between CT- and DWI-ASPECTS against DWI-ASPECTS were performed for each time-window (Fig. 2E,F). In both early- and late-windows, mean bias values for the difference between VIM-ASPECTS (2.06 [95%AI 0.61 to 3.51] and 1.18 [95%AI 0.58 to 1.77]) or NCCT-ASPECTS (3.46 [95%AI 2.08 to 4.83] and 2.23 [95%AI 1.57 to 2.90]) and DWI-ASPECTS trended toward superior accuracy with VIM, however the agreement intervals remained overlapping. Interrater ICCs were similar in the early-window versus the late-window for both VIM and NCCT (ICC = 0.57 [IQR 0.21–0.84] and 0.61 [IQR 0.30–0.84] vs. ICC = 0.65 [IQR 0.32–0.88] and 0.64 [IQR 0.34–0.86]).

## Discussion

In this study, we found that DECT VIM more accurately estimated ischemic core volumes than NCCT or CTP compared to reference standard DWI assessment in AIS-LVO patients. CT remains the mainstay in the imaging triage of suspected AIS-LVO patients for ET, though there have been limited improvements in the non-contrast assessment of core infarction. The increased accuracy of VIM ischemic core delineation over NCCT is of particular clinical relevance given established core volume thresholds in determining ET candidacy^8,9,18,19^. Of note, all CT methods underestimated the ischemic core relative to DWI, which is expected given the greater sensitivity of DWI, however VIM remained the most accurate CT measure examined in this study. While our findings also suggest that VIM more accurately estimated the ischemic core than CTP, commonly applied CBF < 30% segmentation thresholds underestimate the core volume relative to a less restrictive CBF < 38% threshold to reduce false exclusion of patients from ET, which is supported by our adjunct analysis of CBF < 38% volumes, which were similar to VIM derived volumes^14^. In addition, automated segmentation erroneously failed to segment an ischemic core volume for several patients in our cohort with DWI proven core infarcts of ≤ 30 mL, further biasing the analysis. Consequently, we suggest that VIM may be an adjunct to, but should not replace CTP core estimates during AIS-LVO patient triage on the basis of these data alone.

Our analysis of multi-reader ASPECTS determination found that VIM was non-inferior to NCCT, with a trend toward improved qualitative conspicuity of the ischemic core relative to DWI-ASPECTS (mean bias 1.58 vs 2.79). Interrater agreement was moderate for both VIM and NCCT and concordant with previously reported interrater variability in ASPECTS determination^20^. The lack of significant differences in ASPECTS determination and interrater variability in our study suggests that a larger sample size is necessary to determine if the trend toward superior VIM-ASPECTS accuracy is significant beyond known limitations of heterogeneity in ASPECTS interpretation. Nonetheless, our results may remain clinically relevant in risk stratification of AIS-LVO patients, as larger visible ischemic cores assessed by ASPECTS portend a lower likelihood of benefit from endovascular therapy^21^.

Subgroup analyses of AIS-LVO patients presenting in the early- (≤ 6 h) versus late-window (> 6 h) demonstrated superior normalized ischemic core estimates using VIM over NCCT in both early- and late-windows. Bland–Altman analyses indicated significantly greater accuracy of VIM over NCCT core estimation in the early-window (mean bias 0.60 vs. 0.20), but only a trend toward greater accuracy in the late-window (mean bias 0.75 vs. 0.45). As level 1 evidence supporting the use perfusion imaging is currently limited to patients presenting within 6–24 h of LKN, we expect that for the triage of early-window AIS-LVO patients, the superiority of VIM over NCCT in ischemic core determination may better direct early-window treatment decisions^21–23^. The non-significant trend toward greater accuracy of VIM over NCCT in the late-window may be attributed to time-dependent maturation of edema within the infarct, which is concordant with the smaller difference in normalized CT core volumes observed between time-windows^24,25^.

Relative to NCCT, VIM reconstructions qualitatively demonstrate a more uniform attenuation between gray and white matter, with areas of hypoattenuation in acute infarction appearing more starkly contrasted. We hypothesize that the VIM multi-material separation algorithm using hemorrhage, CSF, and iodine definitions generates this homogenized appearance by assuming that individual voxel attenuation must be attributed to the relative contribution of only the base materials, based on the assumption of mass conservation used in the three-material decomposition algorithm^1,26,27^. In these non-contrast examinations, iodine content is zero, therefore the VIM may summarily be considered as a two-material separation algorithm in which the gray and white matter homogenize given the similar attenuation of each to the hemorrhage spectrum, subsequently highlighting parenchymal water along the CSF spectrum, particularly within areas of early developing ionic and cytotoxic edema within the core infarct. This proposed mechanism is compatible with our observation that VIM is relatively superior to NCCT in the early-window, during which time ischemia results in more subtle predominantly ionic and cytotoxic edema^28^. Conversely, in the late-window, vasogenic edema progresses within the maturing ischemic lesion, which may explain the overall greater agreement between VIM and NCCT in predicting the DWI core^28,29^.

Relative to previously described DECT-based edema reconstructions, which performed custom material decompositions using material basis sets involving either air, water, and iodine, or calcium, fat and gray matter, VIM reconstructions are a repurposing of a readily available brain hemorrhage reconstruction module using manufacturer default settings with a material basis set of hemorrhage, water and iodine^5,6^. Our method corroborates prior findings that DECT techniques may improve acute infarct conspicuity, while demonstrating that even a simple, default reconstruction technique is capable of estimating the ischemic core in a carefully selected AIS-LVO population with accuracy comparable to that of perfusion-based estimates and approaching that of DWI, which remain the current reference standards in practice and were not used to estimate true infarct volume in previous studies.

Our study has several limitations. First, we selected only patients in whom there is a matched perfusion profile or who undergo expeditious and complete or near-complete revascularization. Therefore, we assume that the size of the ischemic core from index CT to MRI should minimally change for those with matched infarcts or complete reperfusion, and may modestly progress for those with incomplete reperfusion, but we are unable to assess if progression did in fact occur or if a portion of ischemic core volume differences can be attributed to differences in imaging modality sensitivity. Additionally, our study did not precisely control the time to MRI follow-up or periprocedural variables, introducing an additional bias for patients who did not present with a matched infarct, though for patients who underwent timely and complete reperfusion, we expect core progression to be minimal, regardless of the presenting time-window, while modest growth may be expected for those with incomplete reperfusion^30^. Second, our study may be biased by heterogenous patient treatment and sites of occlusion. Third, ASPECTS assessment is limited by established inter-reader variability, small sample size, and heterogeneity in experience among readers, necessitating further testing to determine if VIM may provide a more reliable basis for ASPECTS determination^20,31^. Fourth, limited utilization of CT for the triage of AIS-LVO patients at our institution and reduced penetration of DECT protocols due to patient motion or body habitus may introduce selection bias. Fifth, our use of automated rather than manual segmentation of CBF < 30% volumes was chosen to avoid manual segmentation errors related to the low-resolution nature of CTP data, but may systematically underestimate the ischemic core volume, limiting the strength of any conclusion comparing VIM to CTP core estimates. Finally, our study uses only a single-vendor DECT scanner and VIM algorithm, which reduces variability in image quality, but limits generalizability. However, VIM may theoretically be reconstructed without regard to vendor, as all DECT are capable of material decomposition and most vendors include comparable default virtual non-contrast decomposition modules.

## Conclusion

We conclude that non-contrast DECT VIM may more accurately estimate the ischemic core than conventional NCCT in AIS-LVO patients, especially in the early-window (≤ 6 h), but expectedly remains modest in approximating the DWI reference standard in ischemia assessment. Currently, AIS-LVO triage is critically reliant upon accurate neuroimaging to appropriately determine treatment candidacy, with CT remaining the most widely available and utilized imaging modality^22,32^. We expect that neurointerventionalists with practices reliant on CT imaging will benefit from the improved infarct detection profile offered by the VIM technique, especially for early-window AIS-LVO patients in whom CTP may not be routinely performed or available. Larger prospective validation of ischemic core estimation by VIM is warranted.

## Supplementary Information

Supplementary Information.

## Abbreviations

DECT: Dual-energy CT
NCCT: Conventional non-contrast CT
VIM: Virtual ischemia maps
AIS-LVO: Acute ischemic stroke due to large-vessel occlusion
CTP: CT perfusion
ET: Endovascular thrombectomy
LKN: Last known normal time
ICC: Intraclass correlation coefficient
TICI: Thrombolysis in cerebral ischemia

## References

[1] Wolman DN, Patel BP, Wintermark M, Heit JJ (2018). Dual-energy computed tomography applications in neurointervention. J. Comput. Assist. Tomogr..

[2] McCollough CH, Leng S, Yu L, Fletcher JG (2015). Dual- and multi-energy CT: Principles, technical approaches, and clinical applications. Radiology.

[3] Grams AE (2018). Improved visualisation of early cerebral infarctions after endovascular stroke therapy using dual-energy computed tomography oedema maps. Eur. Radiol..

[4] Phan CM, Yoo AJ, Hirsch JA, Nogueira RG, Gupta R (2012). Differentiation of hemorrhage from iodinated contrast in different intracranial compartments using dual-energy head CT. AJNR Am. J. Neuroradiol..

[5] Taguchi K (2018). "X-Map 2.0" for edema signal enhancement for acute ischemic stroke using non-contrast-enhanced dual-energy computed tomography. Investig. Radiol..

[6] Mohammed MF (2018). Unenhanced dual-energy computed tomography: Visualization of brain edema. Investig. Radiol..

[7] Lansberg MG (2012). MRI profile and response to endovascular reperfusion after stroke (DEFUSE 2): A prospective cohort study. Lancet Neurol..

[8] Albers GW (2018). Thrombectomy for stroke at 6 to 16 hours with selection by perfusion imaging. N. Engl. J. Med..

[9] Nogueira RG (2018). Thrombectomy 6 to 24 hours after stroke with a mismatch between deficit and infarct. N. Engl. J. Med..

[10] Marks MP (2014). Correlation of AOL recanalization, TIMI reperfusion and TICI reperfusion with infarct growth and clinical outcome. J. Neurointerv. Surg..

[11] Marks MP (2014). Angiographic outcome of endovascular stroke therapy correlated with MR findings, infarct growth, and clinical outcome in the DEFUSE 2 trial. Int. J. Stroke.

[12] Albers GW (2016). Ischemic core and hypoperfusion volumes predict infarct size in SWIFT PRIME. Ann. Neurol..

[13] Heit JJ, Wintermark M (2016). Perfusion computed tomography for the evaluation of acute ischemic stroke: Strengths and pitfalls. Stroke.

[14] Cereda CW (2016). A benchmarking tool to evaluate computer tomography perfusion infarct core predictions against a DWI standard. J. Cereb. Blood Flow Metab..

[15] Wu TY (2016). Software output from semi-automated planimetry can underestimate intracerebral haemorrhage and peri-haematomal oedema volumes by up to 41. Neuroradiology.

[16] Giavarina D (2015). Understanding Bland Altman analysis. Biochem. Med..

[17] Bland JM, Altman DG (1999). Measuring agreement in method comparison studies. Stat. Methods Med. Res..

[18] Saver JL (2015). Stent-retriever thrombectomy after intravenous t-PA vs. t-PA alone in stroke. N. Engl. J. Med..

[19] Campbell BC (2015). Endovascular therapy for ischemic stroke with perfusion-imaging selection. N. Engl. J. Med..

[20] Farzin B (2016). Early CT changes in patients admitted for thrombectomy: Intrarater and interrater agreement. Neurology.

[21] Yoo AJ (2014). Impact of pretreatment noncontrast CT Alberta Stroke Program Early CT Score on clinical outcome after intra-arterial stroke therapy. Stroke.

[22] Powers WJ (2018). 2018 guidelines for the early management of patients with acute ischemic stroke: A guideline for healthcare professionals From the American Heart Association/American Stroke Association. Stroke.

[23] Prakkamakul S, Yoo AJ (2017). ASPECTS CT in acute ischemia: Review of current data. Top Magn. Reson. Imaging.

[24] Broocks G (2018). Quantitative lesion water uptake in acute stroke computed tomography is a predictor of malignant infarction. Stroke.

[25] Hossmann KA (2008). Cerebral ischemia: Models, methods and outcomes. Neuropharmacology.

[26] Liu X, Yu L, Primak AN, McCollough CH (2009). Quantitative imaging of element composition and mass fraction using dual-energy CT: Three-material decomposition. Med. Phys..

[27] Patino M (2016). Material separation using dual-energy CT: Current and emerging applications. Radiographics.

[28] von Kummer R, Dzialowski I (2017). Imaging of cerebral ischemic edema and neuronal death. Neuroradiology.

[29] Minnerup J (2016). Computed tomography-based quantification of lesion water uptake identifies patients within 4.5 hours of stroke onset: A multicenter observational study. Ann. Neurol..

[30] Marks MP (2018). Endovascular treatment in the DEFUSE 3 study. Stroke.

[31] Kobkitsuksakul C, Tritanon O, Suraratdecha V (2018). Interobserver agreement between senior radiology resident, neuroradiology fellow, and experienced neuroradiologist in the rating of Alberta Stroke Program Early Computed Tomography Score (ASPECTS). Diagn. Interv. Radiol..

[32] Campbell BCV (2019). Penumbral imaging and functional outcome in patients with anterior circulation ischaemic stroke treated with endovascular thrombectomy versus medical therapy: A meta-analysis of individual patient-level data. Lancet Neurol..

