# Supplementary material for: Non-contrast dual-energy CT virtual ischemia maps accurately estimate ischemic core size in large-vessel occlusive stroke
Source: Sci Rep. 2021 Mar 24;11:6745. doi: 10.1038/s41598-021-85143-3 (PMC7991428; doi:10.1038/s41598-021-85143-3)
Supplement: Supplementary file 1 — Supplementary Information. [file 41598_2021_85143_MOESM1_ESM.docx]

**Non-Contrast Dual-Energy CT Virtual Ischemia Maps Accurately Estimate Ischemic Core Size in Large-Vessel Occlusive Stroke**

Dylan N. Wolman, MD*^1^, Fasco van Ommen, MS^2^, Elizabeth Tong, MD^1^, Frans Kauw, MD^2^, Jan Willem Dankbaar, MD, PhD^2^, Edwin Bennink, PhD^2^, Hugo W.A.M. de Jong, PhD^2^, Lior Molvin, MBA^3^, Max Wintermark, MD, MBA^1^, Jeremy J. Heit, MD, PhD*^1^

^1^Stanford University Hospital, Department of Neuroimaging and Neurointervention, Palo Alto, CA, USA.

^2^University Medical Center Utrecht, Department of Radiology, Utrecht, Netherlands.

^3^Stanford University Hospital, Department of Radiology, Palo Alto, CA, USA.

**Supplemental Methods**

The Shapiro-Wilk test was performed using the Royston method to assess for volume distribution normality, and a logarithmic regression of the data was performed to correct for non-Gaussian distributions in Bland-Altman plots. Uncorrected Bland-Altman plots are provided in Supplemental Figure 1, and the composing data are described in the results within the main text.

**Supplemental Results**

Shapiro-Wilk testing demonstrated that all volume data were non-normally distributed (VIM: V=3.7, P=0.003; NCCT: V=5.7, P<0.001; DWI: V=8.2, P<0.001), therefore all graphical data presented in the main text is shown following logarithmic correction.

**Supplemental Figure 1**: Patient selection flowchart


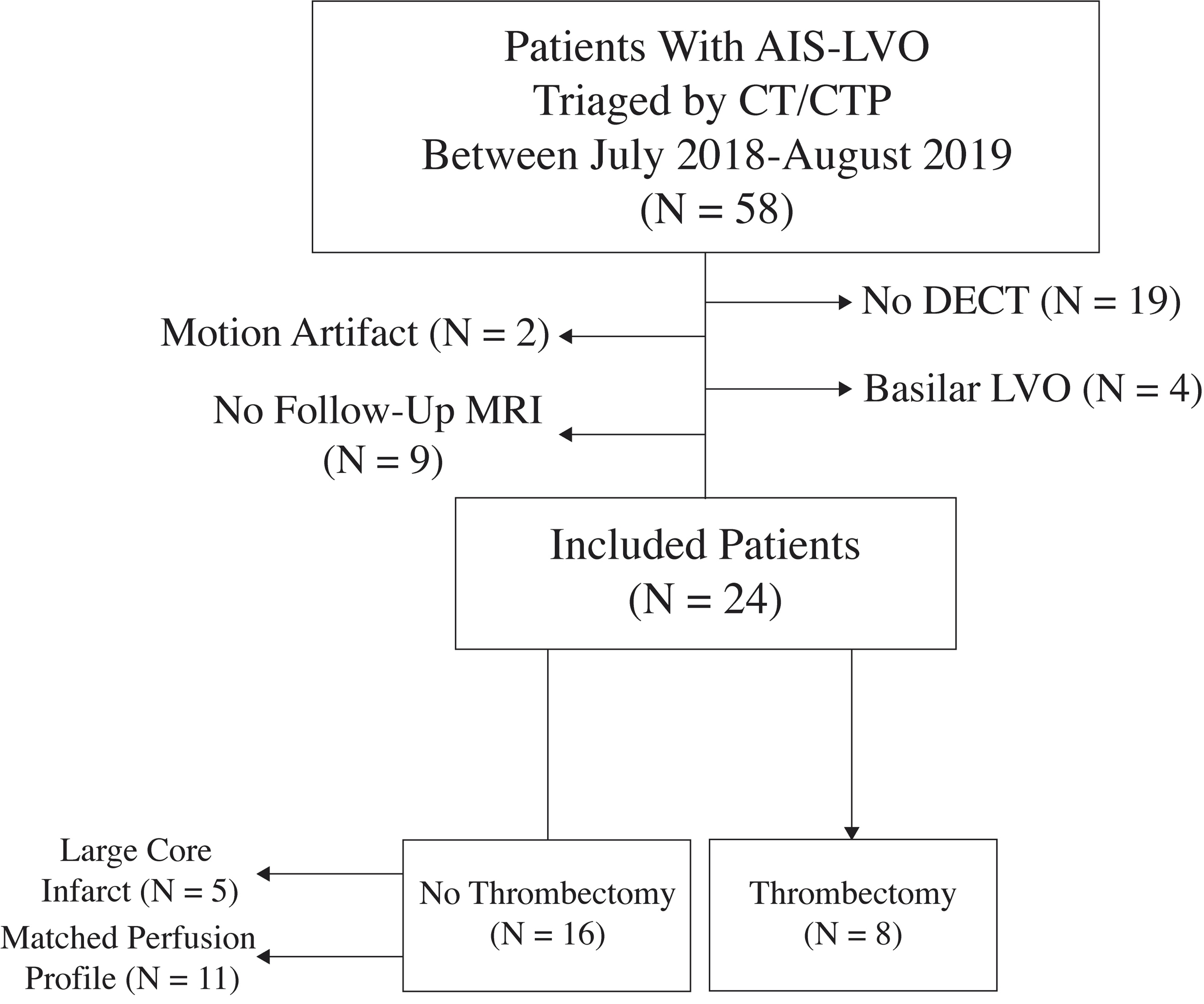


Flowchart demonstrating the method of patient inclusion and exclusion. Consecutive patients undergoing CT and CTP triage with AIS-LVO between July 2018-August 2019 were included, with exclusion of patients with posterior circulation occlusions, those with significantly motion degraded examinations, absent follow-up MRI, or studies performed without dual-energy mode activated. Patients were excluded from subsequent thrombectomy if the region of core infarction was large (≥70 mL) or matched to the region of perfusion deficit (matched perfusion profile).

**Supplemental Figure 2**: Overall and time-dichotomized Bland-Altman analyses of ischemic core volume agreement without logarithmic correction


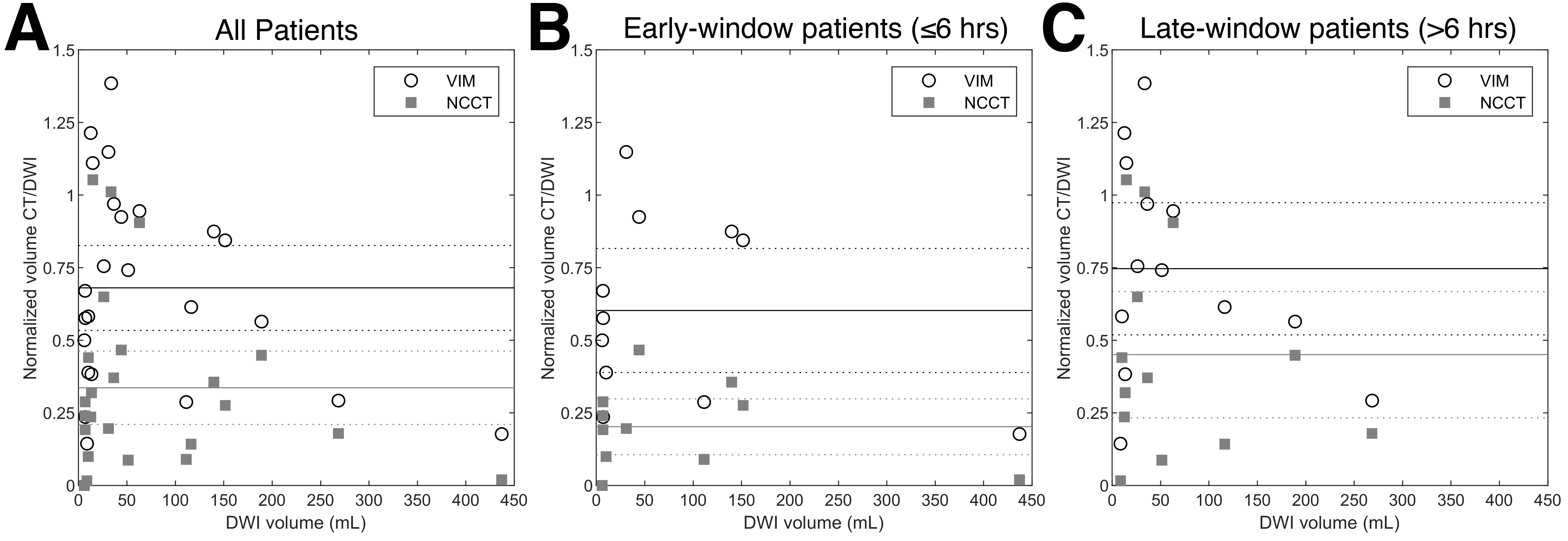


VIM and NCCT volumes normalized against reference DWI volumes for all patients (A) and for patients in the early- (B) and late-windows (C) are plotted against the corresponding DWI volumes, with solid lines indicating the mean bias value for each measurement, and hashed lines indicating the 95% agreement interval limits. Values closer to 1.0 indicate greater agreement with the reference DWI volume.

**Supplemental Figure 3**: Comparison of direct agreement between CT- and DWI-ASPECTS


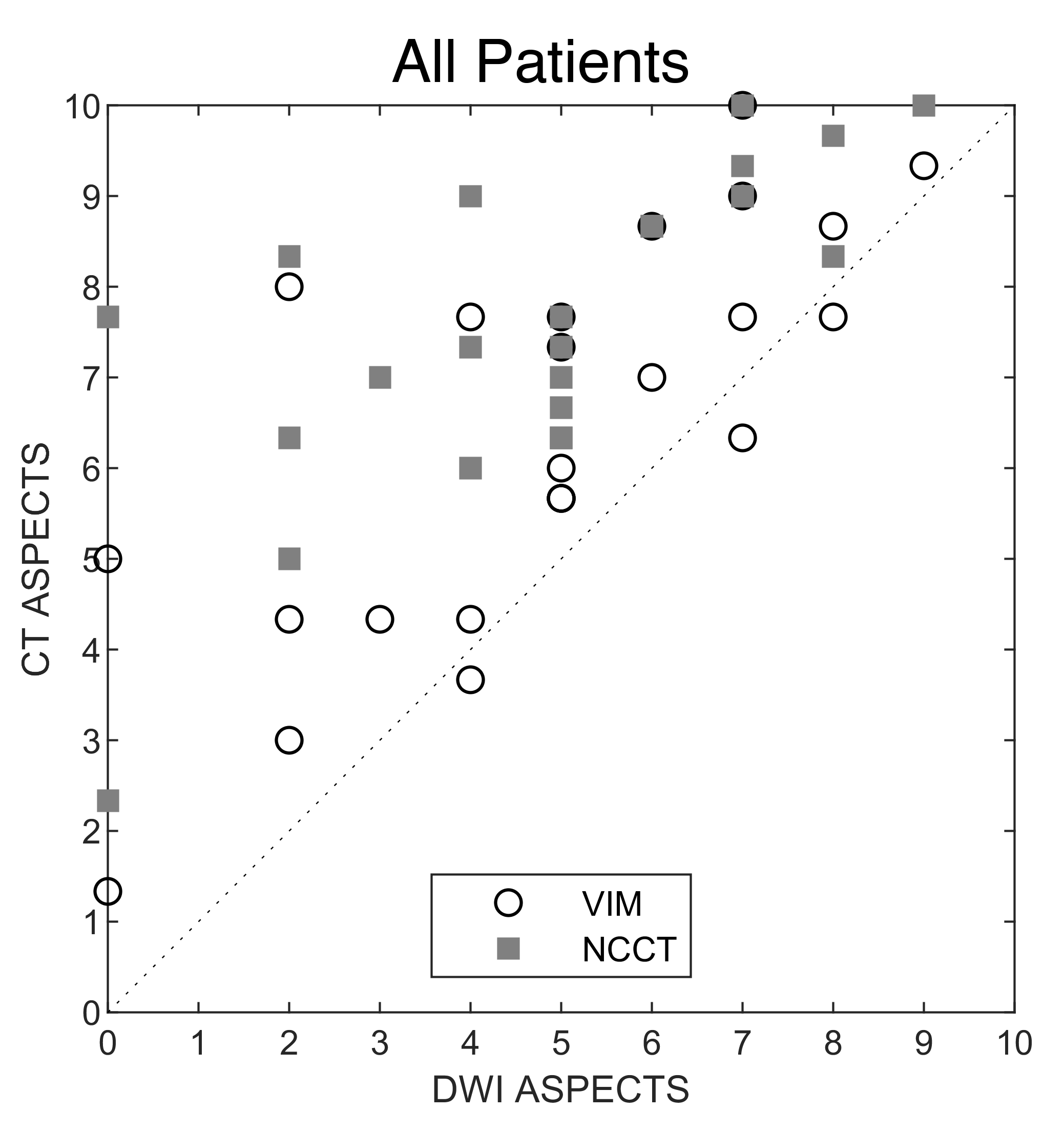


VIM-ASPECTS and NCCT-ASPECTS for all included patients are plotted against the corresponding consensus DWI-ASPECTS, with values falling upon the line of normality (dotted line) indicating the greatest agreement to the DWI reference.

**Supplemental Figure 4**: ASPECTS and ischemic core volume agreement as a function of time


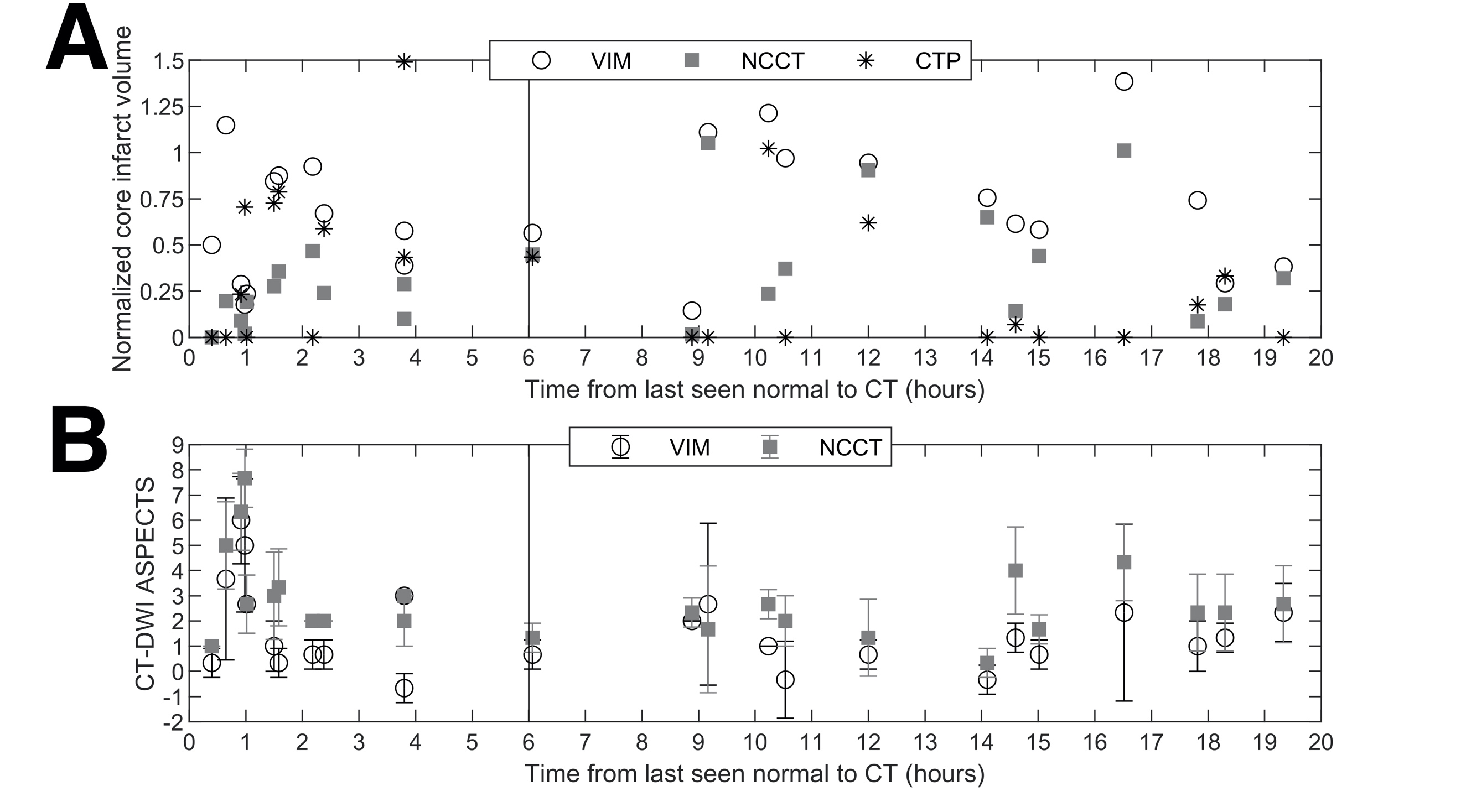


(A) Scatter plot comparing NCCT, VIM, and automatic CBF<30% ischemic core volumes normalized against manual DWI volumes for each case as a function of time since last known normal. Values of 1.0 indicate perfect agreement with the reference DWI volume, while values >1.0 indicate overestimated ischemic core volumes. (B) Scatter plot comparing consensus DWI-ASPECTS subtracted from pooled NCCT- and VIM-ASPECTS for each case as a function of time since last known normal. The degree of agreement between the CT- and DWI-ASPECTS is estimated by decreasing plotted values, with a value of 0 indicating perfect agreement, and values <0 indicating over-estimation of qualitative core infarct extent by the CT score. A vertical line at 6 hours visually separates the early- and late-window patients.

**Supplemental Table 1**: Overall individual reader ASPECTS agreement

| Individual Reader ASPECTS Comparisons | | | | | |
| --- | --- | --- | --- | --- | --- |
|  | NCCT ASPECTS | VIM ASPECTS | P-Value (1) | P-Value (2) | P-Value (3) |
|  | (Accuracy vs. DWI) | (Accuracy vs. DWI) |  |  |  |
| Reader 1 | 7.3±2.2 (68%) | 5.9±2.3 (74%) | <0.001 | <0.001 | <0.001 |
| Reader 2 | 7.6±1.9 (66%) | 6.5±2.4 (72%) | 0.14 | <0.001 | 0.001 |
| Reader 3 | 8.0±2.0 (61%) | 6.8±2.8 (67%) | 0.42 | 0.08 | 0.002 |

Overall individual reader ASPECTS comparisons are presented. Overall individual reader ASPECTS are compared in a pairwise fashion, with each numbered P-value representing a comparison between the given reader’s VIM-ASPECTS and the NCCT-ASPECTS for readers 1, 2, and 3, respectively, with the parenthetical number corresponding to the comparison reader’s number. Individual reader accuracy relative to consensus DWI-ASPECTS is provided parenthetically following each mean CT-ASPECTS, and is calculated as the fraction of the individual reader’s score versus the consensus reference standard DWI-ASPECTS.

**Supplemental Table 2**: Individual reader ASPECTS agreement as a function of time

| Individual Reader ASPECTS Comparisons | | | | | |
| --- | --- | --- | --- | --- | --- |
| Early-Window | NCCT ASPECTS | VIM ASPECTS | P-Value (1) | P-Value (2) | P-Value (3) |
| Reader 1 | 7.9±1.7 | 6.2±2.5 | 0.004 | 0.008 | 0.002 |
| Reader 2 | 8.2±1.8 | 7.3±2.0 | 0.33 | 0.03 | 0.02 |
| Reader 3 | 8.7±1.6 | 7.2±2.9 | 0.49 | 0.27 | 0.02 |
| Late-Window | NCCT ASPECTS | VIM ASPECTS | P-Value (1) | P-Value (2) | P-Value (3) |
| Reader 1 | 6.8±2.5 | 5.8±2.3 | 0.02 | 0.008 | 0.01 |
| Reader 2 | 7.2±2.0 | 5.9±2.8 | 0.31 | 0.004 | 0.04 |
| Reader 3 | 7.4±2.2 | 6.5±2.8 | 0.63 | 0.27 | 0.07 |

Time-dichotomized individual reader ASPECTS are compared in a pairwise fashion, with each numbered P-value representing a comparison between the given reader’s VIM-ASPECTS and the NCCT-ASPECTS for readers 1, 2, and 3, respectively, with the parenthetical number corresponding to the comparison reader’s number.
